# Association Between Hypochromic Microcytic Anemia in Pregnancy and Cord Blood Levels of Cadmium, Lead, Manganese, Mercury, and Selenium

**DOI:** 10.1007/s12011-025-04742-7

**Published:** 2025-07-11

**Authors:** Shannon Isennock, Mohamad Elabiad

**Affiliations:** 1 Department of Pediatrics, Division of Neonatology, University of Tennessee Health Science Center, 853 Jefferson Ave, Memphis, TN 38163 USA; 2 Regional One Health Hospital, 877 Jefferson Ave, Memphis, TN 38103 USA

**Keywords:** Iron deficiency anemia, Pregnancy, Lead, Mercury, Selenium, Cord blood

## Abstract

Iron deficiency anemia (IDA) has been associated with increased blood lead (BPb). Increased BPb in pregnancy has been associated with increased cord BPb. The association between IDA in pregnancy and cord PB levels has not been previously investigated. It is thus hypothesized that IDA in pregnancy results in increased cord BPb levels. Prospectively, cord blood, from term infants delivered in Memphis, TN, was tested for lead, manganese, mercury, and selenium. Maternal charts were retrospectively reviewed and subjects enrolled into two groups: IDA group with hemoglobin < 9 g/dL, 65 fl < MCV 75 fl, MCHC < 32 g/dL and controls with hemoglobin > 12 g/dL, 80 fl < MCV < 95 fl and MCHC > 34 g/dL. Exclusion criteria were chronic conditions, sickle cell disease, and thalassemia. Fifty-five infants were included, with 27 in the maternal anemia group and 28 in the control group. There were no significant differences between groups in maternal age, pregnancy histories, prenatal vitamin and iron intakes, or other morbidities. Pregnancies with anemia had significantly lower BPb and blood mercury (BHg) levels and significantly higher blood selenium (BSe) levels than those without anemia,0.24 (0.18,0.32) µg/L, 0.19(0.17,0.52), 184 ± 31 vs 0.33 (0.27,0.45), 0.55 (0.27,0.92), 160 ± 33, respectively. Conclusion: Pregnancies with anemia were associated with significantly higher cord BSe levels and lower BPb and BHg levels than pregnancies without anemia. Selenium consumption has been shown to lower BPb and BHg levels. This may explain our findings. Future studies are needed to investigate the role of selenium consumption as a protective agent against the transfer of heavy metals across the placenta.

## Introduction

Iron deficiency is a pervasive global health issue and represents one of the most treatable and preventable causes of daily-adjusted life-years lost. Women of reproductive age shoulder a sizeable majority of this burden. Due to high fetal demands, iron deficiency anemia is the leading cause of anemia in pregnancies, complicating as many as 40% of pregnancies worldwide [1]. Anemia during pregnancy has been associated with maternal manifestations like fatigue, tachycardia, and pallor, as well as an increased risk of cesarean delivery [2]. Anemia during pregnancy has also been associated with adverse fetal and neonatal outcomes, including stillbirth and low birth weight [3].

Iron is an essential micronutrient. At the cellular level, the transport of iron is handled by Divalent Metal Transporter 1 (DMT1). DMT1 itself transports seven other different metals, including cadmium, manganese, lead, and zinc [4]. There is also evidence that DMT1 may be involved in the transport of mercury [5, 6]. In iron deficiency, the levels of DMT1 across the gut are increased to enhance iron absorption. If iron availability in the gut remains low, and since DMT1 is a shared transporter, an unwanted consequence occurs where lead absorption increases by several folds [7]. This phenomenon likely extends to pregnancy, where evidence supports an association between IDA and increased blood lead levels in pregnant mothers [8]. In this study, Yadav et al. reported that 11% of women with IDA had elevated BPb levels as compared to only 2.4% of women without IDA. Currently, pregnant and lactating women with a history of high lead levels should be screened and evaluated for IDA. However, there are no recommendations to screen pregnancies with IDA for lead levels [9].

In children, neurodevelopmental delay has been associated with maternal IDA during pregnancy [10]. Similarly, neurodevelopmental delay has also been associated with high maternal lead levels during pregnancy [11]. Although both are likely standalone associations, there is a possibility that the association between IDA during pregnancy and neurodevelopmental delay may be partly potentiated by increased lead absorption.

Selenium, a nonmetal, is an essential element that has many important roles in the body, including roles in thyroid function, DNA synthesis, and the regulation of oxidative stress. When evaluating exposure to heavy metals, selenium is commonly included due to its ability to modify mercury toxicology [12]. There is also recent evidence of a nonlinear association between selenium levels and anemia-related indicators, similar to those used in this study [13].

We thus hypothesized that, compared with pregnancies without IDA, IDA in pregnancy would result in increased maternal blood lead (BPb) levels, reflected in significantly higher cord levels. Our primary objective was to compare cord BPb levels between pregnancies with and without IDA. Our secondary objective was to evaluate the blood levels of four other elements that were concomitantly available for analysis and had a reasonable association with fetal heavy metal toxicity, iron deficiency anemia, and iron status. These were cadmium, manganese, mercury, and selenium.

## Material and Methods

This case–control study started with a prospective sample collection that was followed by a retrospective chart review. First, all leftover cord blood samples during the study period were scavenged from the blood bank. Next, charts of infants with these cord samples were reviewed for enrollment criteria. Lastly, and once infants met inclusion criteria, the charts of their respective mothers were reviewed for maternal enrollment criteria. Data were obtained for infants delivered between December 2021 and February 2022 at Regional One Health, Memphis, TN, USA. This hospital averages between 1200 and 1300 deliveries per year. The Institutional Review Board approved the study protocol at the University of Tennessee Health Science Center.

### Patients

Infant inclusion criteria included a gestational age at delivery of ≥ 37 weeks. Respective charts of enrolled infants were then reviewed to evaluate for maternal exclusion criteria. Infant and maternal data were retrospectively collected through a chart review. Infant data collected included birth weight, gestational age, head circumference, length, gender, and race. Maternal data collected included age, race, BMI, gravida and para status, anemia history, medication intake during pregnancy, with a focus on specific medications like iron and calcium, and home zip code. Maternal laboratory results collected included the predelivery cell blood count with its indices. Group 1 was designated as cases of mothers with iron deficiency anemia. As very few mothers had iron studies available, cases of suspected IDA were used instead. Suspected IDA cases were defined based on blood indices showing microcytosis and hypochromia. These were defined as mothers with a hemoglobin < 9 g/dL, MCV level range of 65 to 75 fl, MCHC < 32.5 g/dL, immediate predelivery WBC count < 10,000, and a Mentzer index ≥ 14 [14]. Group 2 consisted of the controls, who had a hemoglobin level greater than 12 g/dL, an MCV level range of 80 to 95 fl, and an MCHC greater than 34 g/dL.

Maternal exclusion criteria included pregnancies with a suspected or a proven diagnosis of chorioamnionitis or with predelivery WBC ≥ 10,000. Other maternal conditions that were also excluded included sickle cell trait and B-thalassemia trait, tobacco and alcohol consumption during pregnancy, and chronic conditions or illnesses that may interfere with the interpretation of maternal lab profile. Recipients of blood transfusions during pregnancy and infant-mother pairs where the infant receives a diagnosis of early-onset sepsis with > 48 h of antibiotics were also excluded.

### Sample Collection and Analysis

As a standard of practice, cord blood was routinely collected on all deliveries. This was done after placental delivery. After cutting the cord, free-flowing blood would drip into an EDTA-containing tube. Samples were then sent and stored in the blood bank at 4 °C. After 5–7 days, samples to be discarded and never utilized by the blood bank were saved for the study. Blood was gently mixed by inverting the tightly sealed tubes back and forth. A volume of 0.5 mL was then poured into a certified metal-free tube. These samples were shipped to the Centers for Disease Control and Prevention (CDC) for analysis.

The analytical method itself has been previously summarized [15] with full details also available at the CDC’s website [16]. In brief, whole blood samples were analyzed for cadmium, lead, manganese, mercury, and selenium concentrations using inductively coupled plasma triple quadrupole mass spectrometry (ICP-QQQ-MS) method. Prior to analysis, blood samples were prepared by diluting 50 µL of whole blood to 1 mL with a matrix solution containing tetramethyl ammonia hydroxide, 1% ethanol, 0.01% ammonium pyrrolidine dithiocarbamate, 0.05% Triton™ X-100, and a 5 μg/L iridium, rhodium, and tellurium internal standards. The matrix was introduced to an Agilent 8900 ICP-QQQ-MS instrument, (Agilent, Santa Clara, CA, www.agilent.com). Quantification was performed by comparing the observed signal ratio (analyte/internal standard) from the dilution of the patient blood sample to the signal ratio response curve from the working calibrators. The calibration curve *R*2 requirements for all metals were a minimum of 0.98, typically 0.99 to 1.000. The limits of detection in blood for lead, manganese, mercury, and selenium were 0.049 µg/dL, 0.52 µg/L, 0.17 µg/L, and 9.9 µg/L respectively. Cadmium was rarely detected in any of the samples and the levels were insufficient to draw any meaningful conclusions.

### Sample Size Calculation

Local metal exposure data were more available for lead. It was thus used to calculate the sample size. Setting a power at 80%, alpha error at 5%, and to detect a mean lead level difference of 2 µg/dl (SD 2.5) between the IDA and the non-IDA group, and with 20% additional for inadequate samples, a sample size of 30 in each arm was required for a total of 60 infants.

### Statistics

The data were checked for normality using the Kolmogorov–Smirnov test. Parametric data were presented as mean ± standard deviation and non-parametric data were presented as median (interquartile range). An independent samples t-test was used to compare the two groups when the both were normally distributed, and the Wilcoxon-Mann–Whitney test was used when at least one of them was not. Categorical variables were compared using the χ^2^ test, and data were presented as number (percentage). SAS 9.4 (SAS Institute Inc., Cary, NC) was used for the statistical analyses.

## Results

Fifty-five total umbilical cord samples were included in the study: 27 in the sIDA group and 28 in the control group. There were no significant differences between the groups in demographic and clinical variables, except for race. The Hispanic race was predominant in the controls, while the Black race was predominant in the sIDA group (Table 1). Two subjects declined to share their race.

**Table 1:** Baseline demographic and clinical variables differences between the two groups

Demographic and clinical variables	**Control**	**sIDA**	***p*-value**
Age, years	27 ± 7	25 ± 6	0.11^a^
Gravida	2 (1, 4)	3 (2, 4)	0.22^b^
Para	2 (1, 3)	2 (2, 4)	0.18^b^
Race, *n* (Black, Hispanic, White)	13, 12, 1	24, 2, 1	0.006^c^
Prenatal vitamin, % intake	64	65	0.94^c^
Prenatal iron, % intake	19	19	0.95^c^
BMI	34 ± 5	34 ± 9	0.91^a^
Blood calcium level, mg/dL	8.7 ± 0.5	8.5 ± 0.4	0.29^a^
Known high lead zip code, %	29	37	0.5^c^
Fetal sex, % (male, female)	57, 43	48, 52	0.7^c^
Gestational age, weeks	39 ± 1	39 ± 1	0.59^a^
Birth weight, g	3343 ± 413	3347 ± 418	0.97^a^
Head circumference, cm	34 ± 2	34 ± 1	0.84^a^
Length, cm	51 ± 2	51 ± 2	0.54^a^

^a^*p*-value assessed using the independent samples *t*-test

^b^*p*-value assessed using the Wilcoxon-Mann–Whitney test

^c^*p*-value assessed using the χ^2^ test

By design, pregnancies with sIDA had significantly lower hemoglobin, MCV, and MCHC indices (Table 2). There was no significant difference in white blood cell (WBC) count between the groups (Table 2).

**Table 2:** Differences in cell blood count results

Cell blood counts	Control	sIDA	*p*-value
WBC, × 1000/µL	9 (8, 10)	8.8 (6.9, 11.1)	0.63^b^
Hemoglobin, g/dL	12.4 ± 0.8	8.8 ± 1.4	** < 0.001**^a^
MCV, fl	87 ± 3.5	68 ± 4	** < 0.001**^a^
MCHC, g/dL	34.6 (34.3, 34.9)	31.4 (30.6, 31.9)	** < 0.001**^b^
Platelets, × 1000/µL	204 ± 61	256 ± 87	**0.014**^a^
Mentzer index	21 ± 2	17 (15, 18)	** < 0.001**^b^
RDW%	14.1 (13.8, 15)	18.8 (17.8, 20.2)	** < 0.001**^b^

^a^*p*-value assessed using the independent samples *t*-test

^b^*p*-value assessed using the Wilcoxon-Mann–Whitney test

Pregnancies with sIDA had significantly lower lead and mercury levels and significantly higher selenium levels when compared to control pregnancies (Table 3).

**Table 3:** Comparing levels between controls and pregnancies with anemia

Element	Control level	Expected results in cases	Observed results in cases	sIDA level	*p*-value
Blood lead, µg/L	0.33 (0.27, 0.45)	↑	↓	0.24 (0.18, 0.32)	**0.008**^b^
Blood manganese, µg/L	36 (25, 47)	↑	No difference	39 (29, 44)	0.83^b^
Blood mercury, µg/L	0.55 (0.27, 0.92)	↑	↓	0.19 (0.17, 0.52)	**0.002**^b^
Blood selenium, µg/L	160 ± 33	No difference	↑	184 ± 31	**0.007**^a^

^a^*p*-value assessed using the independent samples *t*-test

^b^*p*-value assessed using the Wilcoxon-Mann–Whitney test

Stratifying the data by race showed that in Blacks results mirrored the aggregate data. The sIDA Black group had lower BPb and BHg and higher BSe levels compared to the control group. In Hispanics, the data were not different between the two groups, albeit there were only two subjects in the sIDA group (Table 4). Whites had one subject in each group. In the control group, the levels were 0.22 µg/L, 132 µg/L, 1.5 µg/L, and 234 µg/L, and in the sIDA group, the levels were 0.26 µg/L, 42 µg/L, 0.24 µg/L, and 167 µg/L for BPb, BMn, BHg, and BSe, respectively. When comparing Black controls to Hispanic controls, there was no statistical difference between the two groups in any of the 4 metal levels. When comparing Black sIDA to Hispanic sIDA, only selenium (*p* = 0.08) and manganese (*p* = 0.07) showed a trend towards significance.

**Table 4:** Comparing levels between controls and pregnancies with anemia stratified by race

	Black			Hispanic		
Element	Control level ***N*** = 13	sIDA level ***N*** = 24	***p***-value	Control level ***N*** **=** 12	sIDA level***N*** = 2	***p***-value
Blood lead, µg/L	0.31 (0.28,0.40)	0.24 (0.18,0.32)	0.049^b^	0.45 (± 0.34)	0.18 (± 0.00)	0.27^a^
Blood manganese, µg/L	31 (23,36)	36 (± 12)	0.27^b^	38 (25,59)	49 (± 7)	0.47^b^
Blood mercury, µg/L	0.54 (± 0.29)	0.41 (0.17,0.53)	0.11^b^	0.77 (± 0.73)	0.17 (0.17,0.17)	0.03^b^
Blood selenium, µg/L	165 (± 30)	187 (± 32)	0.07^a^	147 (± 24)	156 (± 10)	0.65^a^

^a^*p*-value assessed using the independent samples *t*-test

^b^*p*-value assessed using the Wilcoxon-Mann–Whitney test

## Discussion

The association between IDA in pregnancy and increased BPb levels in the pregnant mother is well documented [8]. In this study, we hypothesized that a pregnancy with blood work reflecting a microcytic and a hypochromic anemia, markers of IDA, would be associated with increased cord blood levels of lead and mercury when compared with cord blood collected from pregnancies with normochromic and normocytic indices. Contrary to our hypothesis, the results showed lower levels of cord BPb and BHg in pregnancies with anemia. This was surprising, as the criteria chosen to enroll mothers with likely IDA, based on blood indices, while ruling out or excluding other conditions, were appropriate [17]. What is new in our study is the finding of a significant association between microcytic and hypochromic anemia and higher cord selenium levels. This result may potentially explain the lower levels of cord BPb and BHg observed in our study.

Selenium is an essential dietary micronutrient. Its absorption in the gut primarily occurs through specific transport mechanisms involving selenoproteins that ensure that dietary selenium, whether in inorganic forms (selenate or selenite) or organic forms (selenoamino acids), is effectively absorbed in the small intestine and utilized by the body [18]. There is no evidence that selenium is absorbed using DMT1. However, evidence suggests that changes in selenium levels are associated with changes in certain molecules that utilize DMT1, specifically those related to lead and mercury.

Several reports show an interaction between lead and selenium in women with preeclampsia. While pregnancies with higher maternal lead levels have been associated with preeclampsia [19], selenium seems to mitigate these effects [20]. Animal models show that in the presence of suboptimal levels of selenium, lead toxicity is potentiated [21]. Animal models also demonstrate that selenium mitigates lead toxicity by forming complexes with lead that are subsequently excreted through the feces [22, 23]. In summary, there is clinical and animal evidence that selenium mitigates lead toxicity, at least in part through a chelation-like phenomenon. Selenium mitigates mercury toxicity by forming inert complexes, maintaining the function of critical selenoproteins, and enhancing antioxidant defenses. These mechanisms collectively reduce the bioavailability and toxic effects of mercury ffering protection to the organism [24–26]. Mercury toxicity can further be reduced through urinary excretion of selenium-mercury complexes [27]. In our study, the sIDA group had significantly higher selenium than in the control group. This may have resulted in lower mercury levels in the mother’s blood, and consequently lower levels in the cord blood.

In examining the levels of all three elements of interest—lead, mercury, and selenium—compared to previous reports, we note a study that reported cord BPb levels from infants delivered at our hospital along with other local hospitals [28]. Cord blood lead levels were similar, with 0.33 (0.27, 0.45) µg/dL in our study, compared to 0.37 (0.09, 1.8) µg/dL in the study by Rabito et al. This is strong evidence of the internal validity of our study. The Environmental Protection Agency (EPA) suggests a reference level of 5.8 µg/L for mercury in cord blood. Many studies report average levels below this threshold, although there are instances where levels can vary significantly due to local dietary habits or environmental exposures. In our study, the cord BHg levels were in the lower range of the previously reported cord BHg spectrum for epidemiological studies among non-Arctic European and North American populations [29]. Selenium levels in both groups could offer a possible explanation. Although selenium levels were significantly higher in the sIDA group than in the control group, it is worth noting that both groups had higher cord BSe levels than expected. Studies commonly report levels at < 100 µg/L [29, 30],as compared to our levels of 160 and 184 µg/L in the control and sIDA groups, respectively. These higher selenium cord levels, which also reflect a higher maternal BSe level, may have offered fetal protection and reduced fetal cord BPb and BHg levels.

It is not clear why the cord blood in our study had higher selenium levels. Cord BSe levels have been shown to reflect maternal pregnancy levels [30]. In turn, higher maternal levels are likely to reflect higher selenium intake levels. Local environmental testing in Tennessee has demonstrated low selenium levels in water and fish [31, 32]. Thus, without maternal selenium blood levels, it would not be possible to further evaluate this possibility. This is a factor that future studies could easily address. It is also unclear why the sIDA group had significantly higher BSe levels than the controls. Two possible scenarios may explain these results. In the first scenario, similar to how IDA can precipitate increased lead uptake, it is possible that maternal IDA during pregnancy may cause an increase in selenium absorption. There are currently no reports that have described a similar association. Maternal labs would be helpful to explore this further. In the second scenario, we look at the study by Zhou et al., who reported an association between low BSe levels and anemia. Normalization of selenium levels was associated with normalization of blood indices. However, this relation was nonlinear, and as selenium levels increased, individuals developed lower blood level indices like our hypochromic microcytic anemic group [13]. In offering another explanation for our results, we postulate that our inclusion criteria targeting pregnancies with hypochromia and microcytosis as markers of IDA may have retrieved the pregnancies with significantly higher levels of selenium.

Race representation was a significantly different between the two groups with Hispanics predominantly represented in the control group with 12 subjects and only 2 in the sIDA group. Black and Hispanic representation was comparable in the control groups with 13 and 12 subjects respectively. Analysis did not show any difference in metal levels between the two race groups. Hispanics were only represented with two subjects in the sIDA group. Analysis did show a trend towards a difference in manganese and selenium. There could be a real difference between the two groups as there could be different consumption habits due to cultural backgrounds, but this could also be due to the small number of patients or a statistical coincidence due to multiple comparisons.

Our main limitation was the lack of available maternal blood metal levels and iron studies. This was an accepted limitation upfront in this pilot study, as it helped reduce time and logistics to test the hypothesis without significantly compromising the validity of the results. Metal-free collection supplies were not proactively used in the study. Because of the randomness of the left-over sample collections, it is highly unlikely that there were contaminated supplies in one group more than the other. Another important limitation is the inclusion criteria to find pregnancies with IDA. The American College of Obstetrics and Gynecology has published a short list that can be associated with microcytosis [17]. Except for sideroblastic anemia and copper deficiency, all the listed conditions have been ruled out by design or through the study’s results. The retrospective nature of the chart review limited the ability to include complete information about chronic maternal conditions, iron intake, calcium intake, and antenatal vitamin intake including whether selenium supplementation was used. The available chart information, however, did not show any significant baseline characteristics between the groups.

## Conclusion

In conclusion, this study has demonstrated that maternal hypochromic microcytic anemia is associated with significantly lower lead and mercury levels and higher selenium levels in infant cord blood compared to cord blood samples from mothers without hypochromic microcytic anemia. Future studies should evaluate the association of lead, mercury, selenium, and iron studies in the mother-cord blood pair to confirm our findings. Studies should also evaluate the possible role of selenium in mitigating lead and mercury transfer to the fetus during pregnancy.

## Data Availability

No datasets were generated or analysed during the current study.
